# Genetic interaction analysis comes to the diploid human pathogen *Candida albicans*

**DOI:** 10.1371/journal.ppat.1008399

**Published:** 2020-04-23

**Authors:** Virginia E. Glazier, Damian J. Krysan

**Affiliations:** 1 Department of Biology, Niagara University, New York, New York, United States of America; 2 Departments of Pediatrics and Microbiology/Immunology, Carver College of Medicine, University of Iowa, Iowa City, Iowa, United States of America; Geisel School of Medicine at Dartmouth, UNITED STATES

## Introduction

In the last 15 years, mycologists studying human fungal pathogens have seen great improvements in the methods for manipulating the genomes of these organisms. One result of these improvements is that large-scale deletion sets for *Candida albicans* [1], *C*. *glabrata* [2], *Cryptococcus neoformans* [3], and *Aspergillus fumigatus* [4] are available to the communities. This has greatly improved the depth and breadth of single gene–based genetic profiling of these important human pathogens. To date, however, these methods and resources have not been applied to the logical next step: functional genetic interaction analysis using strains with multiple mutations. The power of this approach has been most dramatically demonstrated in the model organism *Saccharomyces cerevisiae* based on the systematic approaches pioneered by Andrews, Boone, and others [5]. Although genetic networks have been constructed for important phenotypes in pathogenic fungi based on single gene analysis, expression profiling, and chromatin immunoprecipitation, the functional significance of such interactions have rarely been tested with double mutants.

The exception to this general statement is the systematic genetic interaction studies of *C*. *albicans* that have been described, recently. In this Pearl, we highlight biological [6], technical [7, 8, 9], and strategic developments [10] that have greatly improved the ability of researchers to generate systematic sets of *C*. *albicans* strains containing deletion mutations at multiple loci. We hope this discussion prompts the more routine use of this powerful genetic tool in the study of pathogenic fungi.

### Principles and rationale of genetic interaction analysis

**Fig 1:**
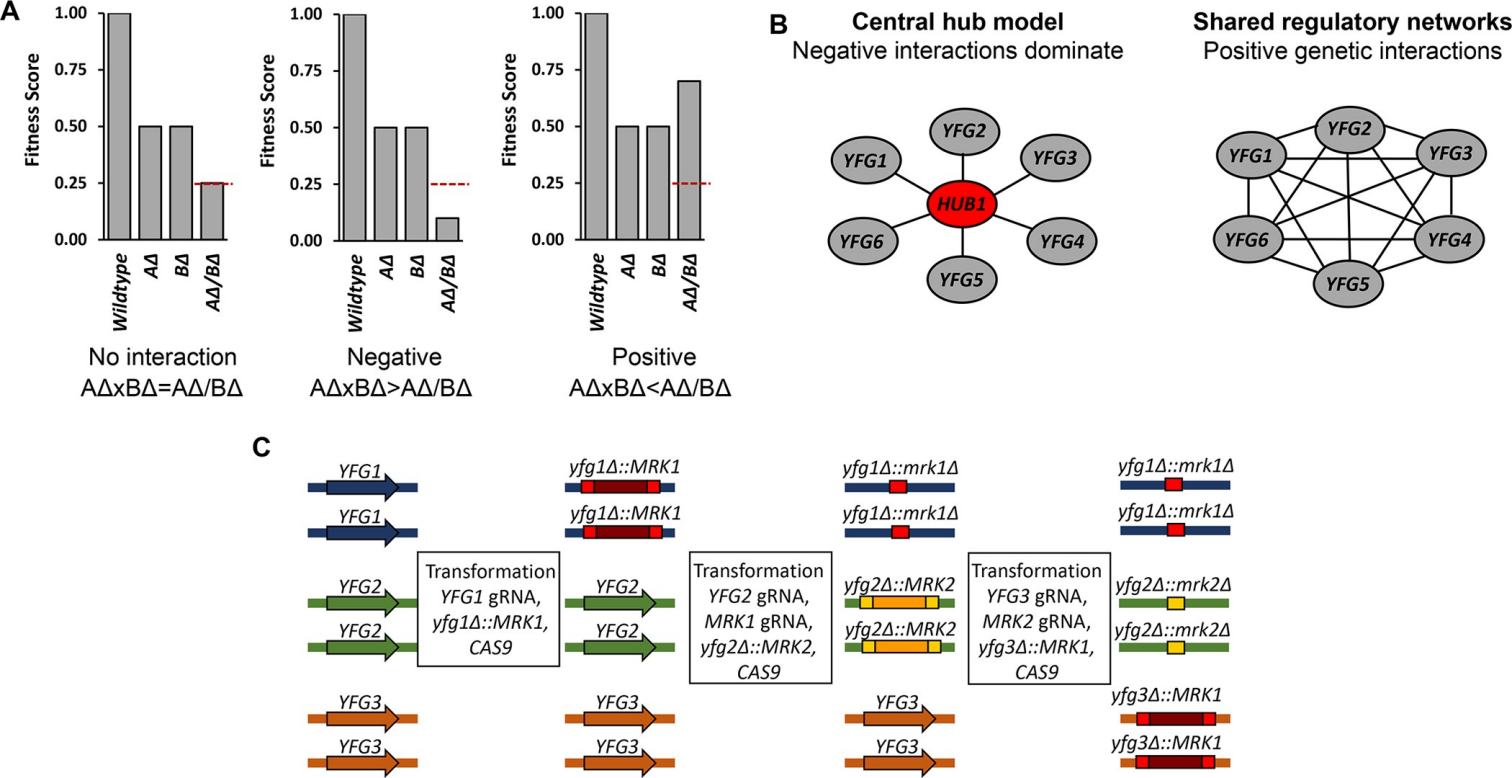
Principles of genetic interaction. (**A**) Definitions of genetic interactions based on the multiplicative model. AΔ and BΔ represent generic mutants. (**B**) Negative genetic interactions of multiple genes (indicated by solid line) with a shared (often essential) gene frequently indicates a hub model. A set of genes with multiple positive interactions (indicated by dashed line) frequently indicates regulatory pathway interactions. (**C**) The CRIME method to generate multiple homozygous deletion mutants in *Candida albicans* [6]. Transformation reactions include Ca*CAS9*, a deletion construct with flanking homologous sequence to deletion site; gRNA for the gene deletion target; and a gRNA for the marker to allow for marker excision and recycling. CRIME, CRISPR-Cas9-induced marker excision; gRNA, guideRNA.

Traditionally, gene function has been inferred from single mutations, yet there are important limitations to this approach. Inherent in the evolution of genomes is the capacity for genetic buffering, which prevents the mutation of 1 gene from causing disruption of function and/or death of the organism [11]. Therefore, the observed phenotype of a single gene mutation is generally a composite of the effect of the mutation itself modulated by genetic interactions. One of the most common approaches to characterizing these interactions is to generate strains pairing a loss of function mutation in the gene of interest with loss of function mutations in candidate interacting genes (**Fig 1A**); the other most common approach is to overexpress candidate interacting genes, but our review will focus on the former approach.

The combination of 2 loss of function mutations results in 3 possible phenotypes (**Fig 1A**): (1) the double mutant phenotype is the simple product of the individual mutations, indicating that the genes do not interact; (2) the double mutant displays a more severe phenotype than the simple product of the individual mutant phenotypes and thus shows a negative interaction (also called synergistic or synthetic); (3) the double mutant is less affected than expected and hence displays a positive interaction (also called suppressive or alleviating). The large-scale, systematic genetic interaction studies in *S*. *cerevisiae* [12, 13] have provided fundamental information about the nature and biological meaning of genetic interactions. For example, an average gene participates in 100 negative interactions and 65 positive interactions (**Fig 1A**). Negative genetic interactions can result if 2 genes carry out redundant functions, are part of a larger protein complex, or are components of a simple regulatory hub (**Fig 1B**). In contrast, positive interactions typically imply that 2 genes function in the same pathway or that the 2 genes are part of a highly interconnected network of genes that buffers a given biological process. Based on these general principles, testable, mechanistic hypotheses regarding the functions of gene pairs and networks can be generated from interaction data.

### CRISPR/Cas9 cuts the time to a *C*. *albicans* double mutant by at least half

The principle barrier to routine genetic interaction analysis in *C*. *albicans* was the simple fact that a total of 4 separate transformations were required to make a double mutant using traditional genetic approaches [14]. Because CRISPR allows one to create a homozygous mutant in a single transformation [7, 8], a double mutant can now be made in half the time using 2 genetic markers instead of 4 (without recycling), using what have become now standard CRISPR methods [15]. The efficiency of gene deletion using CRISPR-Cas9 systems approaches 80% for many loci, making it is also possible to disrupt 2 genes in a single transformation with reasonable efficiency, as described by Nguyen and colleagues [13]. The efficiency of multigene deletions per transformation is the product of each individual gene deletion rate, and thus, triple and quadruple deletions are likely to be difficult to achieve without extensive screening.

Consequently, the ability to efficiently disrupt multigene families will still require marker recycling strategies. CRISPR-based approaches have also led to improvements in this area. In addition to the system developed by Nguyen and colleagues [16], Huang and colleagues [8] have devised an elegant recycling system called CRIME (CRISPR-Cas9 induced marker excision). Following CRISPR-Cas9–induced deletion of a gene (**Fig 1C**), a marker excision CRISPR transformation is conducted to excise the marker from the deletion site. Within the same transformation, a second guide RNA can also be introduced to delete another gene locus, resulting in the simultaneous excision of the initially used marker from the first disrupted gene locus and deletion of the second gene locus with a second marker. Subsequent rounds of marker recycling enable the deletion of multiple genes within a single strain using only 2 marker types. It is also possible to use traditional recyclable markers as repair constructs in CRISPR-based deletion schemes [17].

These methods are expected to greatly improve *C*. *albicans* biologists’ ability to probe the function of families of highly homologous genes. For example, Gonzalez-Hernandez and colleagues used a combination of traditional and CRISPR-based genetics to generate strains lacking multiple homologs of the phosphomannosyltransferase, Mnn4 [18]. They were able to show that although a number of homologs appeared to be partially redundant with Mnn4, it was the predominant phosphomannosyltransferase under the majority of in vitro growth conditions. It would be interesting to see if those functional relationships are also extant during infection.

### Gene drive-based arrays of multiple deletion mutants generated from haploid *C*. *albicans* strains

**Fig 2:**
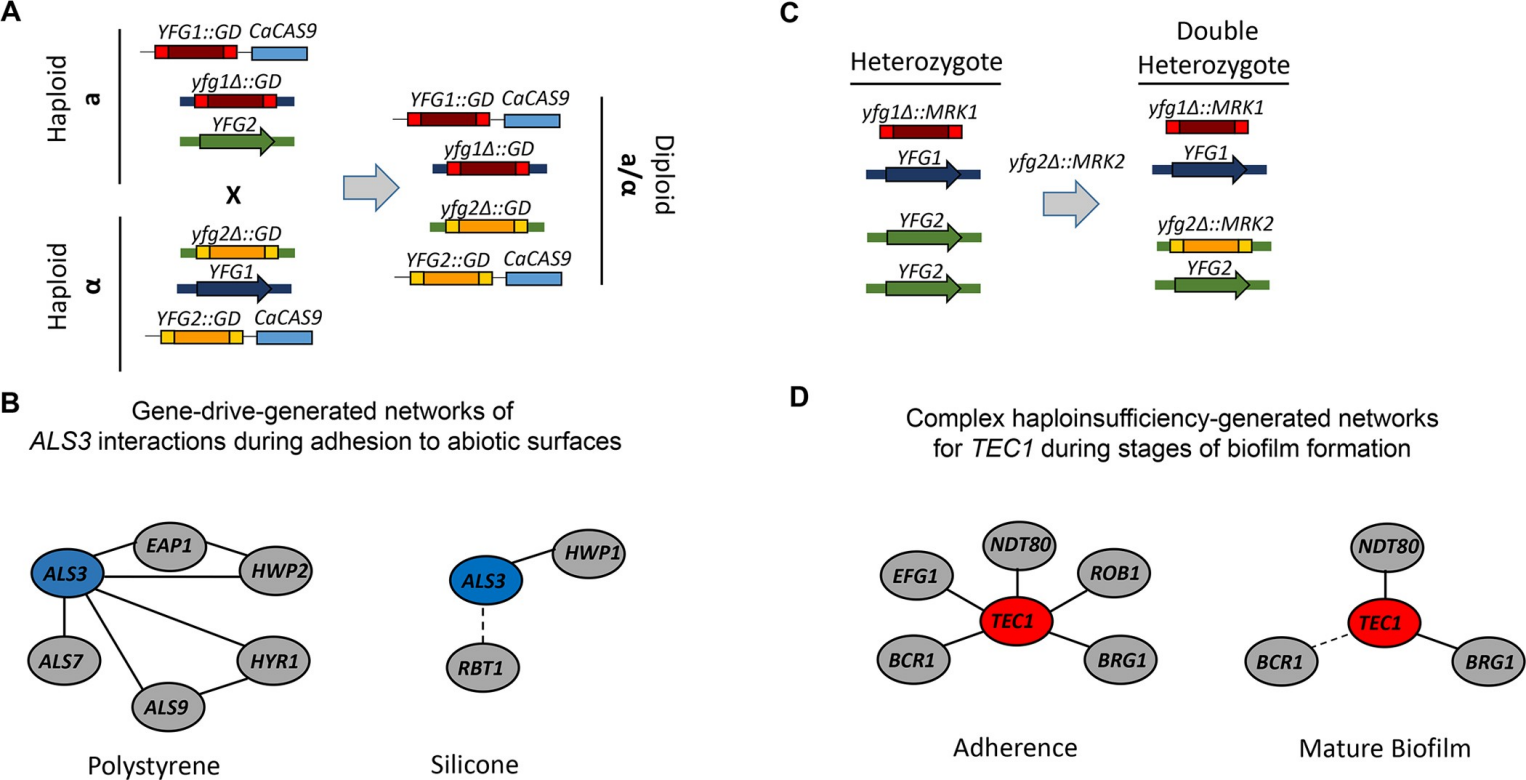
Gene-drive and complex haploinsufficiency-based approaches to systematic genetic interaction analysis. **(A)** Haploid strains of opposite mating types containing a construct harboring *CaCAS9*, *NAT*, and a cassette comprised of guide RNAs with homology sequences for either *YFG1* or *YFG2* are inserted into the putatively empty *NEU5L* intergenic region. The haploid a (*yfg1*Δ) and haploid α (*yfg2*Δ) are mated. The diploid contains both cassettes, and thereby, homozygous deletions strains are isolated. **(B)** Shapiro and colleagues [7] developed the platform and showed that the genetic interactions of the adhesion *ALS3* vary with the type of surface to which *C*. *albicans* is adhering (negative interaction indicated by solid line, and positive interaction indicated by dash). **(C)** Generation of double heterozygote strains using standard homologous recombination driven *C*. *albicans* gene disruption. **(D)** *TEC1* functions as a co-regulatory hub in the adherence step of biofilm formation but has a very different set of interactions at the mature biofilm stage. *C*. *albicans*, *Candida albicans*.

Recently, Shapiro and colleagues [9] reported a creative approach to the systematic generation and study of large sets of double homozygous deletion mutants by taking advantage of the recent discovery of a stable haploid form of *C*. *albicans* [6]. In this approach, genes are disrupted in a haploid strain using an integrated cassette encoding Cas9 and guide RNAs for that gene. The haploid strain is then mated with another haploid strain containing a second gene disrupted with guideRNAs (gRNAs) (Fig 2A); the incoming gRNAs will mediate disruption of the respective alleles provided by the opposite mating type. Importantly, wild-type reference strains generated from the less fit haploid parents retain their virulence in mice. As Shapiro and colleagues reported [9], X x Y matrices of double mutants for large gene families such as drug efflux pumps and adhesins can be systematically generated and studied. An informative observation from their initial report is that the network of adhesins required for *C*. *albicans* to adhere to abiotic surfaces varies with the exact chemical nature of the surface (Fig 2B). These data nicely highlight the power of this approach, particularly in the setting of analyzing multigene families with potentially redundant functions.

### Complex haploinsufficiency

The methods described in the previous sections are used to generate *C*. *albicans* strains with homozygous deletions in multiple genes. Essential genes are not amenable to these approaches. It is important to keep in mind that essentiality does not just apply to viability. For example, many *C*. *albicans* transcription factors (TFs) are absolutely essential for important processes such as biofilm formation and hyphal morphogenesis [19, 20]. If a double homozygous mutant is constructed with such a TF, then only positive genetic interactions will be measurable. We and others have shown that double heterozygous mutants can be used to identify genetic interactions by virtue of a phenomenon called complex haploinsufficiency (CHI) [10, 21].

CHI is observed when a strain with heterozygous mutations in 2 different loci (Fig 2C) shows a phenotype relative to the single mutants that is consistent with a genetic interaction (Fig 1C). We used a CHI to identify genetic interactions among a set of 6 TFs that are required for biofilm formation (Fig 2D); because deletion of any of the TFs essentially abolished biofilm formation, double homozygous mutants would not provide useful information [20, 21]. From this set of data, we identified a new function for *TEC1*. During the adherence step of biofilm formation [10], *tec1*ΔΔ has no phenotype, but *tec1*Δ negatively interacts with all 5 biofilm TF heterozygous deletion mutants. Thus, *TEC1* plays an important co-regulatory function during adherence that would not be evident from single gene analysis. At the mature biofilm stage, interestingly, *TEC1* interacts with only 3 of the other TFs [21], indicating that the functional relationships between this network of TFs are dynamic and dependent on the stage of biofilm formation. CHI, therefore, can be a useful complement to the study of homozygous deletions, particularly when homozygous deletions are either lethal or completely abolish the phenotype of interest.

### Beyond single gene analysis in *C*. *albicans*

The genetic analysis of *C*. *albicans* has identified many genes that contribute to important virulence-related phenotypes, such as hyphal morphogenesis and biofilm formation. For example, at least 20 TFs have a reduced ability to form hyphae in *C*. *albicans* [1]. At this point, however, we know very little about how those TFs work together to regulate. Genetic interaction analysis as described earlier will allow those relationships to be defined functionally. Our initial studies in this area indicate that specific TFs work with others and that these relationships are dynamic [10]. As demonstrated by our CHI experiments [10, 21] and the double mutant array approach of Shapiro and colleagues [9], modestly sized, focused arrays of strains containing systematic deletions of pairs of genes with previously characterized functions can provide new insights into genetic network function without recourse to large scale sets of mutant arrays. Thus, genetic interaction analysis does not need to be genome wide to be informative. Now, thanks to the tools and approaches summarized previously, *C*. *albicans* genetic interaction analysis can be brought into the mainstream. It is our hope that other pathogenic fungi will follow.
